# The Royal Society RAMP modelling initiative

**DOI:** 10.1098/rsta.2021.0316

**Published:** 2022-10-03

**Authors:** G. J. Ackland, J. Panovska-Griffiths, W. Waites, M. E. Cates

**Affiliations:** ^1^ Institute of Condensed Matter and Complex Systems, School of Physics and Astronomy, University of Edinburgh, Edinburgh EH9 3FD, UK; ^2^ The Big Data Institute, Nuffield Department of Medicine, University of Oxford, Oxford OX1 4AW, UK; ^3^ The Queen’s College, University of Oxford, Oxford OX1 4AW, UK; ^4^ Department of Computer and Information Sciences, University of Strathclyde, Glasgow G1 1XH, UK; ^5^ DAMTP, University of Cambridge, Cambridge CB3 0WA, UK

**Keywords:** epidemics, mathematical modelling, COVID-19

## Abstract

Normally, science proceeds following a well-established set of principles. Studies are done with an emphasis on correctness, are submitted to a journal editor who evaluates their relevance, and then undergo anonymous peer review by experts before publication in a journal and acceptance by the scientific community via the open literature. This process is slow, but its accuracy has served all fields of science well. In an emergency situation, different priorities come to the fore. Research and review need to be conducted quickly, and the target audience consists of policymakers. Scientists must jostle for the attention of non-specialists without sacrificing rigour, and must deal not only with peer assessment but also with media scrutiny by journalists who may have agendas other than ensuring scientific correctness. Here, we describe how the Royal Society coordinated efforts of diverse scientists to help model the coronavirus epidemic.

This article is part of the theme issue ‘Technical challenges of modelling real-life epidemics and examples of overcoming these’.

Most of the work in this issue was carried out or supported by a Royal Society initiative, Rapid Assistance in Modelling the Pandemic (RAMP) [1].

The coronavirus pandemic created a unique situation for science, with scientific modelling becoming a tool for developing informed policy advice on the control of the spreading virus. But this also created an environment where work had to be done very quickly, initially based on limited data, whilst remaining robust, correct and transparent. Traditional methods of science became unfeasible for delivering science on the necessary time scale: different approaches were necessary for research, funding, recruitment, training, working conditions and reviewing both the methods and the outcomes.

In February 2020, the UK already had a number of strong, established epidemiology groups and an independent national advisory body, Scientific Pandemic Influenza Group on Modelling (SPI-M), which had most of the expertise required. However, the scale of the modelling challenge was far in excess of what the existing epidemiology groups were set up to deal with.

RAMP was a novel and experimental way of organizing science during an emergency. Designed and led by scientists, it operated on a volunteer basis without a budget. To use a military analogy, RAMP recruited a volunteer army to supplement the professional regulars. Around 2000 responses, from individuals to sizeable research teams, responded to the initial call for computer-literate volunteers [2].^1^

The most pressing concern was to augment the existing groups, adapting and accelerating existing code to the specific needs and datastreams associated with COVID-19. Much of this day-to-day work did not require previous epidemiological experience. Overall, RAMP provided more than 15 person-years of postdoctoral-level support to the existing UK epidemiology community. Since this occurred during lockdown with work from home, there was no additional HR or infrastructure cost and most of the volunteers were able to redeploy research computing resources to modelling the epidemic.

Another challenge was dealing with the deluge of unrefereed preprints which appeared describing work on the pandemic. RAMP adopted a two-level approach to this task. The ‘crowdsourced review system’ was based on a bulletin board set up by EPCC at the University of Edinburgh (the RAMP Forums). This allowed all RAMP volunteers, among others, to post unsolicited reviews of preprints. Unlike conventional reviewing, this process was transparent and turned out to be generally more constructive than is often the case in traditional publishing; this could be due to the fact that reviewers themselves chose papers they were interested in reviewing. Most of these reviews were by experienced reviewers from outside the epidemiological community, who were able to assess the scientific methodology and statistical significance of the claims made. By this stage, everyone was also well acquainted with what was important for policy. Alongside the Forums, but separate from them, RAMP introduced a Rapid Review system, which functioned essentially like a journal review system.^2^ This sourced peer review in the traditional journal style for preprints passed upwards from the ‘crowdsourced review’ or downwards from SPI-M, Public Health England and UKHSA. Preprints were reviewed for both correctness and relevance to the ongoing epidemic. Significant code reviews were also undertaken by this route.

Peer review has always been an unrewarded activity for academics, but the character of the emergency, combined with the convening power of the Royal Society, meant that it proved easy to achieve a rapid turnaround of reviewing.

Given the scale of the response to RAMP, there were many more willing volunteers than the existing groups could assimilate. So a secondary theme within RAMP was ‘New Modelling’. The led to some significant codes developed from scratch: from Mathematics in Cambridge, a compartment model code/framework, PyRoss [3]; from Physics in Durham, an agent-based model for the UK at the scale of individual people, JUNE [4]; from Edinburgh, a data-driven *R*-calculation and medium-term prediction code [5]; and from Computer Science at Strathclyde, an application of process calculus to couple a within-host immune-response model to a population-level epidemic [6]. A pan-Scotland collaboration of animal disease modellers, the Scottish COVID-19 Response Consortium, produced an entire suite of codes and linking data pipelines [7].

A final strand was the provision of software engineering to rewrite, speed up, perform reproducibility, validation and verification tests, and create efficient workflows for existing codes [8]. This included agent-based models such as CovidSim [9–12] and Covasim [13].

RAMP also coordinated modelling expertise for work outside epidemiology, in areas such as fluid dynamics of viral dispersal, movement of people through buildings, comorbidities and software validation. In these fields, as well as in the New Modelling activity, there was a significant rate of participation from non-academic modellers, based in organizations ranging from merchant banks to engineering firms to football clubs.

A central lesson from RAMP is that there exists, right across the UK, a latent capacity of thousands of volunteers who can be deployed on a time scale of days in an emergency situation, so long as structure for organizing them can be created fast enough. These were experienced people, almost all PhDs, willing to work to tight deadlines while furloughed or in their spare time. The status of the Royal Society placed it in a unique position as an apolitical ‘honest broker’—perhaps the one organization in the UK capable of inspiring so many scientists to work for free at short notice.

COVID-19 was a unique crisis, but there will be future emergencies requiring rapid response. Thirty-eight possibilities were laid out in 2020 in the UK National Risk Register [14]; for all of these accurate, impartial review of data will be essential, and for many of them detailed modelling will be crucial. One can imagine internet vulnerabilities, space-weather events, nuclear disaster, or escape of biological and chemical agents. These low-probability, rapid-onset events provide a different type of challenge from ongoing crises such as climate change or poverty, and call for an entirely different model of response.

Succinctly put, with regard to dealing with emergencies: ‘The problem in society we have at the moment is everyone is afraid for making a mistake, everyone is afraid of the consequence of error but the greatest error is not to move.’^3^ There are structural reasons why institutions can be slow to respond to novel crises. The people within these institutions have to operate within constraints designed mainly for stability in normal times. An honest mistake can be career-ending.

Volunteers do not generally have these constraints; they have much greater freedom of action. Not only do volunteers bring valuable outside perspectives, but this freedom means that their response can be very agile. The challenge is coordination. We do not know in advance who will present themselves to help or what kind of help will be needed for a particular emergency. We do know that people will emerge with knowledge, skills and abilities, and the willingness to assist. What is needed is an interface—a way to make a collection of individual volunteers understandable to institutions that are accustomed to working with established entities rather than *ad hoc* groupings. This is a pattern that plays out repeatedly in crises.^4^ In this case, the Royal Society provided such an interface for the modelling community. The need to create institutional interfaces in this way in response to an emergency in order to make effective use of agile volunteer response is a key lesson from this pandemic.

## Data Availability

This article does not contain any additional data.

## Footnotes

1 The call for volunteers was coordinated by Kostas Kavoussanakis of EPCC at the University of Edinburgh.

2 This work was led by Alan Goriely, Philip Maini and Robin Thompson.

3 Dr Mike Ryan, WHO Executive Director for the Health Emergencies Programme, at a press briefing in March 2020, referring, mainly, to institutional responses to the then emerging pandemic.

4 One of the authors experienced this in the response to Hurricane Katrina in 2005, for example.
